# Staging laparoscopy and Pressurized IntraPeritoneal Aerosol Chemotherapy (PIPAC) for peritoneal metastasis: safe access to the abdomen

**DOI:** 10.1515/pp-2019-0004

**Published:** 2019-03-21

**Authors:** Torben Glatz, Philipp Horvath, Sven A. Lang, Rami Archid, Giorgi Nadiradze

**Affiliations:** Department of General and Visceral Surgery, University of Freiburg, Freiburg, Germany; Department of General and Transplant Surgery and National Center for Pleura and Peritoneum, University of Tübingen, Tübingen, Germany

**Keywords:** abdominal access, chemotherapy, laparoscopy, peritoneal metastasis, PIPAC

## Abstract

**Background:**

Pressurized IntraPeritoneal Aerosol Chemotherapy (PIPAC) is an innovative drug delivery technique. Most common indication is palliative therapy of peritoneal metastasis of gastrointestinal and gynecological origin in the salvage situation. Access to the abdomen is the critical step of the procedure, since most patients had previous surgery. Potential pitfalls include non-access because of adhesions, bowel access lesions and postoperative subcutaneous toxic emphysema.

**Methods:**

We propose a technique, the “finger-access technique” that might prevent largely these pitfalls. A minilaparotomy of 3 cm is performed in the midline, a finger introduced into the abdomen and a 5-mm double-balloon trocar (no Hasson trocar) is placed under finger protection at some distance of the first incision. The fascia of the minilaparotomy, not the skin, is then closed. The abdomen is insufflated with CO_2_ and tightness is controlled with saline solution in the minilaparotomy. A second 10–12 mm trocar is then introduced under videoscopic control. The first trocar is then visualized through the second one to exclude a bowel lesion during first access.

**Results and conclusions:**

In our hands, this access technique has shown to be safe and effective.

## Introduction

At least 50 % of laparoscopic complications occur during the initial entry into the abdomen [1]. Complications are rare but can be severe, including vascular or bowel injury [2]. In patients with peritoneal metastasis, diagnostic laparoscopy is perceived as a challenge due to a hostile abdomen, increased risk due to prior surgeries, incomplete assessment or tumor recurrence at the port sites. However, in a retrospective multi-institutional study on 217 consecutive patients, laparoscopic access to the abdomen was possible in 92.5 % patients. The incidence of bowel access lesions was 0.4 %, and no postoperative port-site recurrence was reported [3]. These data suggest that laparoscopy is feasible and safe in patients with peritoneal metastasis.

Pressurized IntraPeritoneal Aerosol Chemotherapy (PIPAC) is an innovative drug delivery technique. Most common indication is palliative therapy of peritoneal metastasis of gastrointestinal and gynecological origin in the salvage situation. PIPAC consists of two procedures: a staging laparoscopy followed by the application of a therapeutic aerosol under pressure into the closed abdomen. Since most patients had previous surgery, access to the abdomen is the critical step of PIPAC, as for staging laparoscopy. In a systematic review, access of the abdomen was possible in 89.1 % patients, depending on patient selection and surgical skills [4]. The non-access rate was higher after cytoreductive surgery and peritonectomy and HIPEC than after other surgeries. In most PIPAC series, bowel access lesions were rare (0–2 %) [5, 6, 7] . However, in a single cohort, incidence of bowel lesions during PIPAC was reported to be as high as 5.7 % [8].

We propose an access technique to the abdomen for performing staging laparoscopy and PIPAC in patients with peritoneal metastasis having undergone previous surgery.

## Materials and methods

### Technique

**Figure 1:**
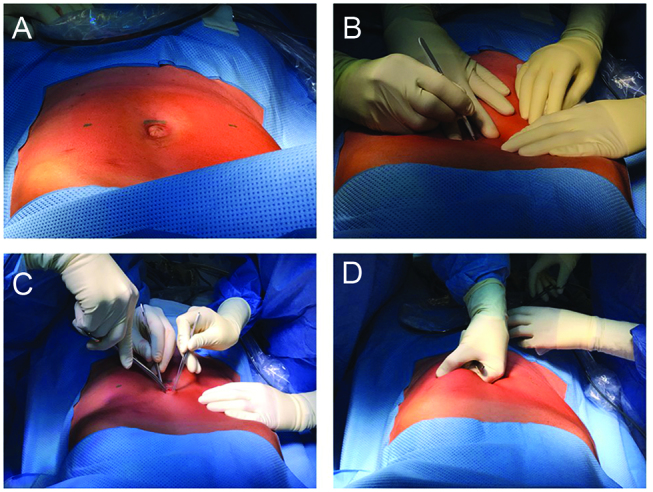
A minilaparotomy of 3 cm is performed in the midline (Panels A, B, C) and a finger introduced into the abdomen (Panel D).

The abdomen is disinfected and draped for a laparotomy. A minilaparotomy of 3 cm is performed, usually in the midline. Then, a finger is introduced into the abdomen and glided laterally along the anterior abdominal wall (Figure 1). Presence of adhesions can be detected by continuously groping the posterior sheet of the rectus abdominis muscle).

**Figure 2:**
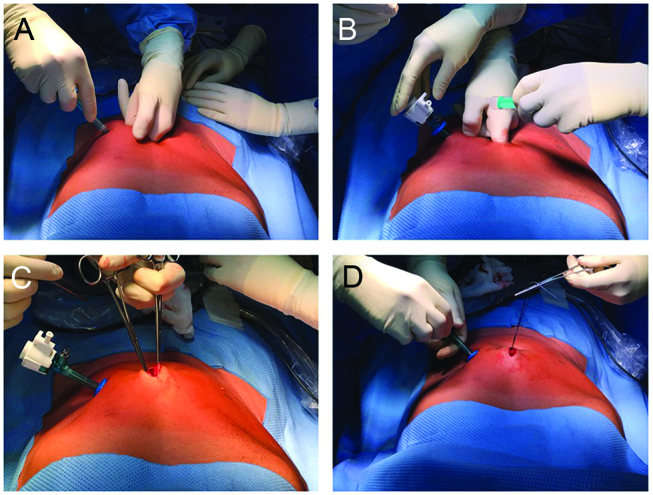
A 5-mm double-balloon trocar (no Hasson trocar) is placed under finger protection at some distance of the first incision, lateral to the epigastric vessels (Panels A and B). The fascia of the minilaparotomy, not the skin, is then closed (Panels C and D).

A 5-mm double-balloon trocar (e. g. Kii^®^, Applied Medical, Düsseldorf, Germany or LapWorks, caMed, Farnham, UK) is placed under finger protection at some distance of the first incision, lateral to the epigastric vessels. The fascia of the minilaparotomy, not the skin, is then closed (Figure 2).

**Figure 3:**
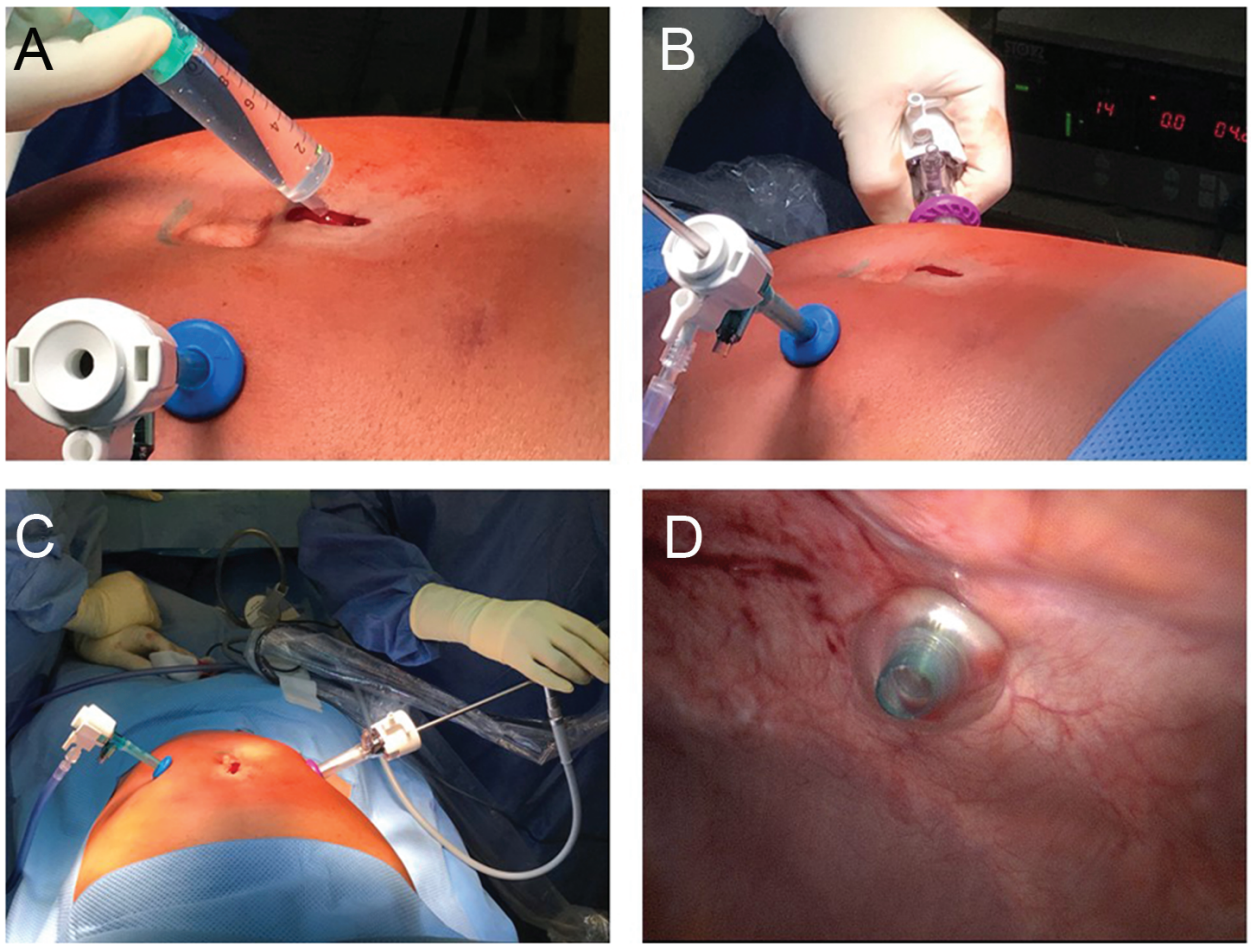
Tightness of the minilaparotomy is verified by filling up the wound with saline, after insufflation of the abdomen (Panel A). After introduction under videoscopic control of a second trocar (Panel B), the intraabdominal position of the tip of the first trocar is visualized in order to exclude any bowel lesion (Panels C and D).

The abdomen is insufflated with CO_2_ and tightness is controlled with saline solution in the minilaparotomy. CO_2_-bubbling documents incomplete closure and an additional stitch has to be placed on the fascia. A 5-mm Hopkins-optic is introduced through the trocar. A second 10–12 mm trocar is then introduced safely under videoscopic control. Then, the camera is introduced into the 10–11 mm, second trocar. The first trocar is visualized to exclude a bowel lesion during first access. (Figure 3).

## Results and discussion

Prior work has shown several techniques to be suitable to access the abdomen for laparoscopy. These techniques include CO_2_-insufflation with Veress needle, open-access with Hasson trocar, optical trocars [3], single-port [9] and combinations of these techniques. According to a recent Cochrane review, evidence is insufficient to support the use of one laparoscopic entry technique over another and many studies excluded patients with previous abdominal surgery [10].

In the USA, the technique used for entering the abdomen for staging laparoscopy in peritoneal metastasis was the open access with Hasson trocar in 57 % patients, optical trocar in 38 % and Veress needle in 5 % [3].

In Germany, Pabst and Tempfer prefer the open access with Hasson trocar to access the abdominal cavity when performing a PIPAC procedure [11]. They place the first trocar in a pararectal position in the left upper quadrant. Reasons for this choice include.

– an optimal angle (45° angle) for aerosolizing the underlying peritoneum.
– a longer spraying distance between the nozzle head and the small bowel
– less interference with enteroparietal adhesions in patients with prior median or subcostal transverse incision.

The authors insist on immediate placement at the beginning of the procedure of 2 stitches on the fascia to guarantee tightness and to prevent leakage of the toxic aerosol with possible infiltration of the subcutaneous tissue. During PIPAC, drugs (such as anthracyclines) are used that are feared for inducing tissue necrosis in the case of extravasation [12]. Thus, it is potentially hazardous to achieve tightness with skin closure rather than fascial closure, since intraabdominal pressure might then force the toxic aerosol into the subcutaneous tissue [11]. In practice, after insufflating the intraabdominal balloon, the surgeon should pull the trocar upwards until a resistance is encountered. This resistance is the proof that the trocar’s balloon is in contact with the abdominal wall. Then, the silicone cone of the Hasson trocar should be glided firmly into the wound and locked in this position in order to guarantee tightness at the fascial, not the skin layer.

In France, Alyami and Eveno prefer to place the trocars into the midline [13, 14]. Rationale is to give a chance to patients with peritoneal metastasis for *en-bloc* resection during secondary cytoreductive surgery, in the case of later development of a port-site recurrence. This is indeed a sound idea for staging laparoscopy before cytoreductive surgery. However, after PIPAC, only a minimal number of patients might eventually benefit, since the incidence of port-site recurrences is very low (between 0–2 %) [6] and only 5 % of these patients will eventually become candidate for secondary cytoreductive surgery after PIPAC [15]. Thus, only 1:1000 to 1:10.000 patients might have a benefit of midline access Moreover, in our experience with staging laparoscopy after prior midline laparotomy, the risk of enteroparietal adhesions is maximal along the scar, which might increase the risk of bowel access lesion [10].

The use of optical trocars for accessing the abdomen has been developed primarily for metabolic surgery. Inserting an optical trocar off the midline 15–18 cm below the xiphoid process has been shown to provide reliable, safe access in the morbidly obese patient, with excellent visualization of the target anatomy [16]. In the context of peritoneal metastasis, optical trocars offer an attractive alternative to open access, since a surgical incision of the fascia is not needed. For example, optical trocars (in combination with Veress needle) have been used in the majority of patients with mucinous ascites without trocar-related complications [17]. In an US cohort, optical trocars have been used for abdominal access in 38 % patients with peritoneal metastasis [3]. However, in our bi-institutional series of 265 consecutive PIPAC between 6/2016 and 2/2019, we have three cases of bowel injury during access (1.1 %), one with optical trocar and two punctions during insufflation with Veress needle [5], but no one with the “finger access” technique. One lesion had to be sutured, chemotherapy was not delivered, there was no postoperative complication. Veress needle access is used in only every 20th patient for insufflating the abdomen after prior surgery [3]. The non-access rate might be higher with this method of insufflation than with open access [10].

In Italy, Vaira et al proposed single-port access for performing PIPAC [9]. For this purpose, the authors propose a 5-cm minilaparotomy in the midline. Then, a single-port device (e. g. QuadPort+, Olympus Medical, Tokyo, Japan) is placed into the minilaparotomy. Twenty-nine PIPAC procedures were performed in 17 patients. Access to peritoneal cavity was possible in all cases and there was no bowel access lesion. Tightness of the abdomen was achieved in all patients. Potential advantages over multiple trocars technique are a lower non-access rate, a lower risk of bowel lesions and a better tightness of the abdomen but this should be confirmed in adequately powered controlled studies.

In any case and without respect of the access technique, it is advisable to insert the camera into each trocar in order to check the correct setting of the second trocar, in particular to exclude any unnoticed iatrogenic bowel lesion.

Patient selection can also help to prevent bowel access lesions. Implementing a PIPAC program is feasible but is associated with a risk of postoperative morbidity, even in teams highly experienced in Management of peritoneal metastasis and requires a learning curve in patient selection. Clearly, the risk of access injury increases with the number of previous surgical procedures and with the presence of dilated bowel loops caused by intestinal (sub)occlusion. The risk for iatrogenic bowel lesions is also higher in the presence of abdominal wall infiltration and/or tumor-associated adhesions. Thus, it has been suggested that PIPAC might be contraindicated in patients in whom clinical examination of the abdomen revealed a rigid and coarse abdominal wall as a sign of a large tumor burden of the visceral and peritoneal peritoneum [11]. When patients are properly selected and the center has gained enough experience, it has been proven possible to perform hundreds of consecutive PIPAC without any adverse event greater than CTCEA 2 [11].

In conclusion, we propose a technical improvement of the open abdominal access for laparoscopy in patients having undergone previous surgery. In our experience, this technique (“finger-access” technique) is feasible and safe for accessing the abdomen for PIPAC. This feasibility study is not designed and not intended to show superiority of the finger-access technique over existing techniques. Further comparative studies with sufficient sample size and adequate statistical power are still needed to prove superiority of a particular access technique.
